# A Major Milestone for the National Children’s Study

**DOI:** 10.1289/ehp.12416

**Published:** 2009-01

**Authors:** Peter Scheidt, Michael Dellarco, Allen Dearry

**Affiliations:** National Children’s Study, Eunice Kennedy Shriver National Institute of Child Health and Human Development, National Institutes of Health Department of Health and Human Services, Bethesda, Maryland; National Institute of Environmental Health Sciences, National Institutes of Health Department of Health and Human Services, Research Triangle Park, North Carolina, E-mail: dearry@niehs.nih.gov

In January 2009, after 8 years of planning and development, the National Children’s Study (NCS; http://www.nationalchildrensstudy.gov/Pages/default.aspx) will begin enrollment and data collection at the first of the initial, or Vanguard, locations, in Duplin County, North Carolina, and in Queens, New York. In spring 2009, the remaining five Vanguard locations will join them, followed by 29 additional locations in 2010 and more in the following 2 years.

The NCS, a national longitudinal cohort study of 100,000 children to be followed from early pregnancy to adulthood, will examine how environmental exposures, interacting with genetic factors and medical access, affect children’s health and development. Beginning enrollment caps a remarkable journey from a bold vision to a reality that was sometimes unpredictable and cloaked in uncertainty.

The NCS began after assertions from both the President’s Task Force on Environmental Health and Safety Risks to Children and the Children’s Health Act of 2000 that a multiagency consortium should carry out a large longitudinal study. Since its inception, the planning and development of the NCS have been led by the National Institute of Child Health and Human Development, along with a consortium of lead agencies that includes the National Institute of Environmental Health Sciences, the Centers for Disease Control and Prevention, and the U.S. Environmental Protection Agency. With broadly defined environmental exposures and all of children’s health and developmental outcomes of concern, the NCS calls for equally broad input by scientific experts, communities, and involved groups. To meet this need, the study has committed to gathering the best input possible through its Federally Chartered Advisory Committee; Interagency Coordinating Committee; over 20 working groups representing > 2,500 clinicians, scientists, and community members; public, peer, and agency reviews; and active participation by the network of Study Centers across the country.

The NCS provides opportunities for related investigations by affiliated and nonaffiliated investigators through adjunct studies conducted at NCS study locations or through analysis of data collected in the NCS to address a wide range of additional questions beyond the core study hypotheses. The study can be expanded to cover even broader maternal, fetal, and child health research. The processes for thoroughly considering the development of methods, proposed changes, and ongoing input are being established through working teams composed of scientists at the various Study Centers, federal agencies, and elsewhere. These teams are organized according to the various issues being addressed in the NCS, such as genetics/epigenetics and genomics/epigenomics, health disparities, environmental exposure, neurodevelopment and cognition, nutrition, and physical/clinical measures.

The nationally representative sampling strategy is probably the most unique and demanding challenge. A representative sample offers the best opportunity to apply the findings of the NCS to all children in the United States. Enrolling and retaining a representative sample of participants will require full engagement of all of the communities involved. Thus, this national study must also become a community study at the level of participating neighborhoods and counties, with input and ownership by community advisory boards, community organizations and institutions, and local citizens.

In spite of the size and the breadth of the NCS, it cannot meet all of the needs and expectations of its supporters. A sample with 100,000 subjects will, by its very size and cost, limit the depth and complexity of many measures. The sample size may prohibit many specialized procedures and technologies, and many questions that investigators want to ask will also be beyond the reach of this study.

The advantages of the NCS that other studies cannot do are its capacity to address compelling problems that are uncommon and to examine interactions between various environmental exposures and genetic factors that require a large sample and detailed measures of multiple exposures and outcomes across life stages. The capability to study possible effects and relationships of many exposure factors on multiple outcomes provides both considerable cost-effectiveness and research opportunities not otherwise available. Access to the necessary resources will be an ongoing challenge, dependent upon many factors, especially the ability of the NCS to fulfill its promise.

Findings from the NCS will ultimately benefit all Americans by providing researchers, health care providers, and public health officials with information from which to develop prevention strategies, health and safety guidelines, and possibly new treatments and cures for disease. As we mark the true beginning of this ambitious study, it is appropriate to recognize that the NCS belongs to all of us—scientists, communities, and families. Realizing its goals will require our continued effort, investment, and ownership.

## Figures and Tables

**Figure:**
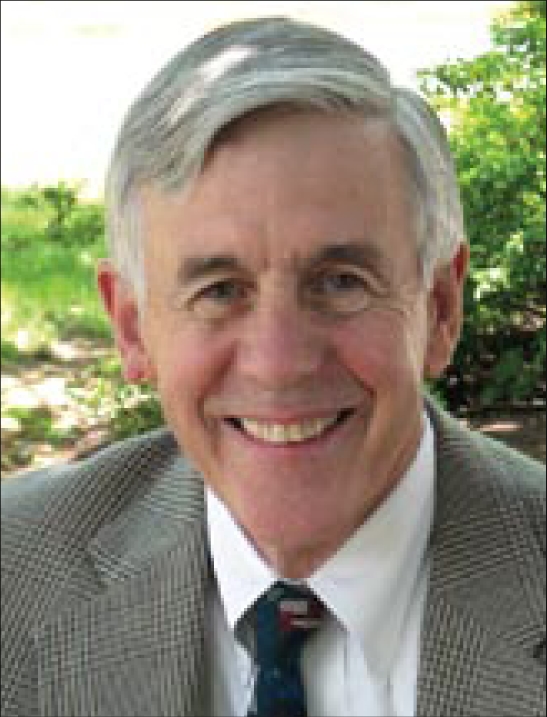
Peter Scheidt

**Figure:**
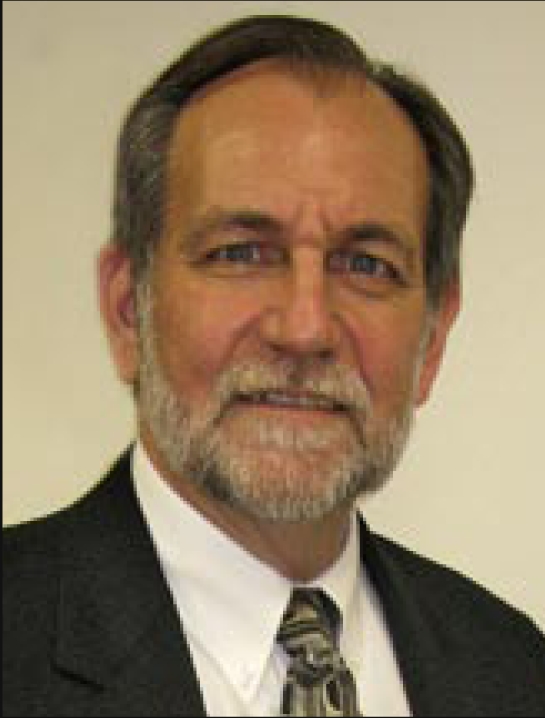
Michael Dellarco

**Figure:**
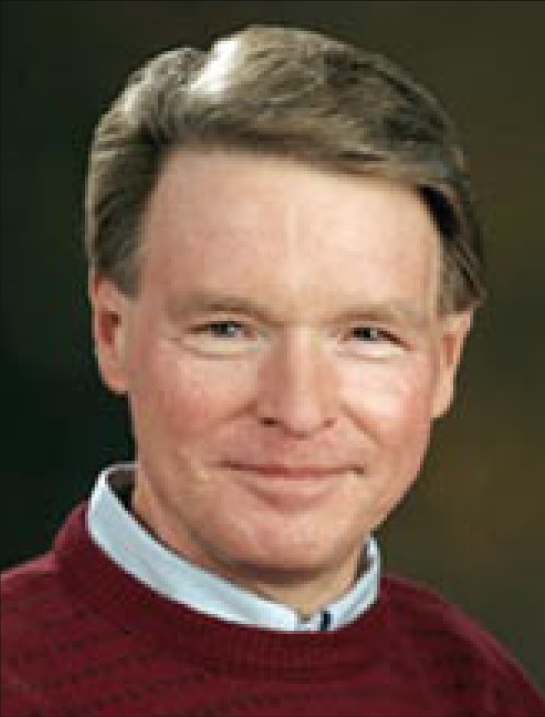
Allen Dearry

